# A three‐lncRNA expression signature associated with the prognosis of gastric cancer patients

**DOI:** 10.1002/cam4.1047

**Published:** 2017-04-26

**Authors:** Peng Song, Bo Jiang, Zhijian Liu, Jie Ding, Song Liu, Wenxian Guan

**Affiliations:** ^1^ Department of General SurgeryNanjing Drum Tower HospitalThe Affiliated Hospital of Nanjing University Medical School321 Zhongshan RoadNanjing210008China

**Keywords:** Gastric cancer, LncRNA, prognosis

## Abstract

Long noncoding RNAs (lncRNAs) have emerged as essential players in gene regulation. An ever‐increasing number of lncRNAs has been found to be associated with the biogenesis and prognosis of gastric cancer (GC). We aimed to develop an lncRNA signature with prognostic value for survival outcomes of GC. Using an lncRNA mining approach, we analyzed the lncRNA expression profiles of 492 GC patients from the Gene Expression Omnibus (GEO), which consisted of the GSE62254 set (*N* = 300) and the GSE15459 set (*N* = 192). The associations between the lncRNAs’ expression and survival outcome were evaluated. A set of three lncRNAs (*LINC01140*,* TGFB2‐OT1*, and *RP11‐347C12.10*) was identified to significantly correlate with overall survival. These lncRNAs were then combined to form a single prognostic signature. Based on this three‐lncRNA expression signature, patients in the GSE62254 set were classified into high‐ and low‐risk subgroups with significantly different overall survival (hazard ratio [HR] = 1.93, *P* < 0.001) and disease‐free survival (HR = 1.91, *P* < 0.001). Good reproducibility for the prognostic value of this lncRNA signature was confirmed in the GSE15459 set. Further analysis showed that the prognostic value of this signature was independent of some clinical characteristics. Gene set enrichment analysis indicated that high‐risk scores positively related to several molecular pathways of cancer metastasis. Our results suggest that this innovative lncRNA expression signature may be a useful biomarker for the prognosis of patients with GC based on bioinformatics analysis.

## Background

Gastric cancer (GC) was the fifth most common malignancy and the second leading cause of cancer‐related death worldwide and was responsible for approximately 1 million new stomach cancer cases and 700,000 deaths in 2012 1. In spite of the improvements in chemotherapy, radiotherapy, and surgical techniques, the 5‐year overall survival (OS) and disease‐free survival (DFS) rate of GC patients remain unsatisfactory. The reasons for these low survival rates include patients who are diagnosed at an advanced stage, thus missing the best opportunity for curative surgery, and cancer recurrence, especially peritoneal recurrence 2. In clinical practice, the American Joint Committee on Cancer (AJCC) TNM staging system is widely used for prognostic prediction. Currently, great efforts are being made to study the biological properties of GC, and there have been many improvements in treatment measures. However, a lack of predictive indicators and therapeutic targets is responsible for the poor outcome. Therefore, a better understanding of the pathogenesis and identification of novel promising prognostic molecular biomarkers are essential for effective therapies, which can contribute to improving the quality of life and survival of GC patients.

Over recent decades, comprehensive investigations of gene regulation in biology have mostly concentrated on protein‐coding genes and their critical genome alterations in the pathogenesis of GC 3, 4. Nevertheless, protein‐coding genes only account for <2% of the whole genome sequence, and the remaining noncoding genes are transcribed into noncoding RNAs (ncRNAs). The ncRNAs are divided into two primary categories according to their size: small ncRNAs (18‐200 nucleotides, e.g., microRNAs and small interfering RNAs) and long noncoding RNAs (lncRNAs, >200 nucleotides) 5. Emerging evidence has indicated that ncRNAs play an important role in cancer pathogenesis, which could provide new insight into GC biology 6, 7. Recently, microRNAs have moved to the forefront of lncRNA GC research, whereas the role of lncRNAs is emerging, with an increasing number of lncRNAs being reported as associated with GC tumorigenesis. Aberrant lncRNA expression has been found in many types of cancers, such as GC 6, esophageal cancer 8, and liver cancer 9. LncRNAs have attracted major attention due to their essential regulatory functions in cell proliferation, migration, and apoptosis. For GC, a substantial portion of lncRNAs is expressed specifically, which suggests their potential role as possible biomarkers and may be predictive of the clinical outcome.

Currently, the methodology of repurposing frequently used microarray data for expression profiling of ncRNAs has been well established 10, 11. For instance, Hu et al. used a series of microarray datasets to build a resource of clinically relevant lncRNAs and found a tumor‐specific prognostic lncRNA signature in colorectal cancer 10. We initially explored previously published gene expression microarray data of large GC cohorts from the Gene Expression Omnibus (GEO) and constructed lncRNA profiles using the abovementioned mining method. Based on the sample‐splitting method and Cox regression analysis, we tried to identify useful lncRNAs associated with the survival of GC patients. The critical goal of this study was to discover key lncRNAs that can act as novel biomarkers to determine GC prognosis.

## Materials and Methods

### GC data sets

The GC gene expression data used in this study were obtained from publicly available GEO databases. To evaluate the association between lncRNA expression signatures and GC survival, we selected the microarray expression profiles based on three criteria: (1) the profiles should be generated by the Affymetrix HG‐U133 Plus 2.0 Array (GPL570 platform), (2) the corresponding clinical data, such as histological classification and follow‐up information, were available online, and (3) the sample size was >100. This resulted in two sets (GSE62254 and GSE15459) that were screened in our study.

### Microarray expression processing and lncRNA profile mining

Raw CEL files of the expression for the two GEO datasets were downloaded, and the Robust Multichip Average (RMA) algorithm was performed for background‐adjustment, quantile normalization, and log‐transformation by the R package “affy” 12. LncRNA profiles were achieved by Seqmap V1.0.8 on a local computer 13. Briefly, the probe sets of Affymetrix HG‐U133 Plus 2.0 were retrieved from the Affymetrix website (http://www.affymetrix.com). We then re‐mapped those probes to the chromosomal positions of the ncRNAs derived from GENCODE (release 24, GRCh38) with no mismatch 14. A total of 2380 probes and 2118 corresponding ncRNA genes were obtained. When multiple probes mapped to the same ncRNA, we used the arithmetic mean of the probe intensities.

### Gene set enrichment analysis (GSEA)

Gene set enrichment analysis (GSEA) was performed using GSEA software V2.2. The gene set used for the enrichment analysis was “c2.cp.v5.0.entrez.gmt” (1330 gene sets), which are canonical representations of biological processes. The GSEA results were visualized in Cytoscape software V3.2.1 using the Enrichment Map plug‐in 15. Gene sets with a false discovery rate (FDR) value <0.05 after performing 1000 random sample permutations were termed “enriched.”

### Statistical analysis

The correlation of lncRNA expression with patients’ OS or DFS was assessed by a univariate Cox regression analysis along with a permutation test using Biometric Research Branch‐Array Tools V4.1.1 (FDR <0.05, *P* < 0.01) 16. Genes were considered statistically significant with a permutation *P* < 0.01. A random survival forests (RSF) variable hunting algorithm was undertaken to further identify valuable lncRNAs 17. In the RSF model, the number of Monte Carlo iterations (nrep) was set as 100, and the value controlling the step size used in the forward process (nstep) was set as 5. Because the GSE62254 set offered a larger sample size and more detailed clinical information than the GSE15459 set, we chose the GSE62254 to determine the risk score formula using a multivariable Cox regression model for the selected lncRNAs. The formula was established by including the expression of these selected lncRNAs, weighted by their estimated regression coefficients. According to this risk score formula, patients in each set were classified into a high‐ or low‐risk group by using the median risk score as the cutoff point. The Kaplan–Meier method with a log‐rank test was adopted to evaluate the survival differences between the low‐ and high‐risk groups. Hazard ratios (HRs) and 95% CIs were estimated by univariate or multivariate Cox regression analysis. Multivariate Cox stepwise regression analysis was employed to determine predictive factors for GC prognosis, with a significance level of *P* < 0.05 for entering and *P* > 0.10 for removing the respective explanatory variables. Receiver operating characteristic (ROC) curves were used to compare the sensitivity and specificity of the prognosis of the lncRNA risk score. Mann–Whitney *U* tests (continuous variables) or chi‐squared tests (categorical variables) were employed to evaluate the associations between lncRNA risk scores and patients of different clinical features. All of the statistical analyses were performed using the R V3.1.3 program (www.rproject.org) and SAS software V9.1. A two‐sided *P* < 0.05 was considered statistically significant.

## Results

### Characteristics of the two GC sets

Two independent sets of GC subjects were included in the present study. The GSE62254 set contained 300 GC patients and had a mean follow‐up time of 50.6 months (range: 1.0–105.7 months). There were 199 males (66.3%) and 101 females (33.7%), 32 (10.7%) cardia patients and 268 (89.3%) noncardia GC patients, and 134 (44.7%) patients with diffuse type and 146 (48.7%) with intestinal type. In addition, 10.0%, 32.0%, 31.7%, and 25.7% of patients were identified as TNM stage I, II, III, and IV, respectively. For the GSE15459 set, 95 patients (49.5%) died of disease‐related to GC in a period of up to 157.8 months (mean: 38.4 months) of follow‐up. There were 125 males (65.1%) and 67 females (34.9%), and 75 (39.1%) diffuse type and 99 (51.6%) intestinal type GC. Additionally, 16.1%, 15.1%, 37.5%, and 31.3% of patients were diagnosed as stage I, II, III, and IV disease, respectively. The demographics and some clinical characteristics of the patients in these two sets were quite similar (*P* = 0.779 for sex, *P* = 0.191 for Lauren classification), whereas the proportion of stage III/IV patients in the GSE15459 set (68.8%) was larger than that in the GSE62254 set (57.3%, Table S1). As two patients in the GSE62254 set did not have definite TNM stage, they were further excluded in the subgroup analysis by TNM stage.

### Identification of prognostic lncRNAs

**Figure 1:**
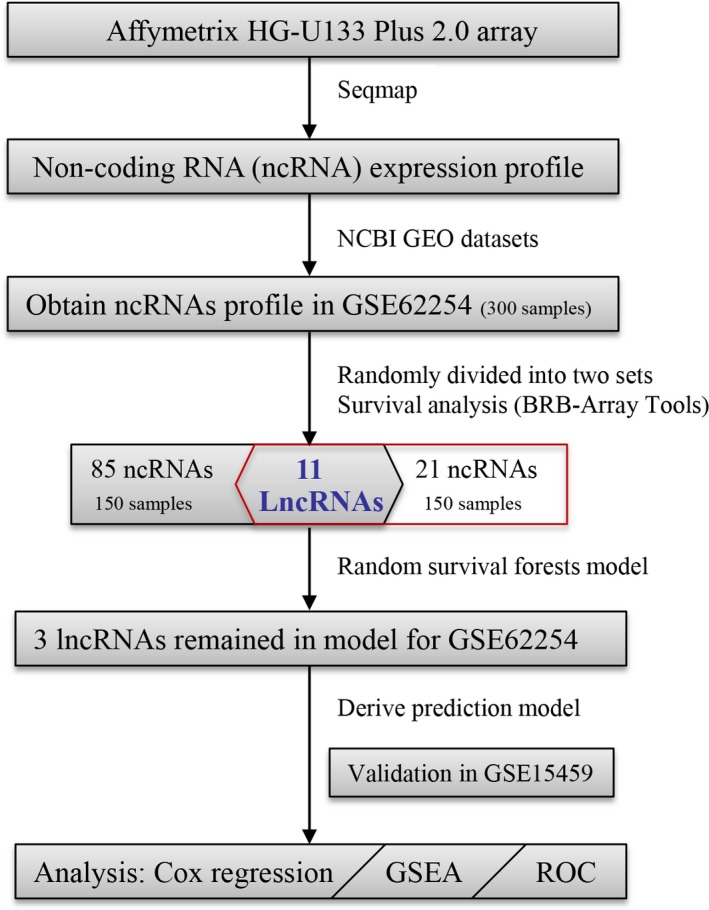
The flowchart of analyses to establish the risk score model and test its predictive value. GEO, Gene Expression Omnibus; GSEA, gene set enrichment analysis; ROC, receiver operating characteristic.

As depicted in Figure 1, the 300 GC patients in GSE62254 were randomly divided into two sets, and both of the GC sets were used for the detection of prognostic ncRNAs. After subjecting the ncRNA expression data to univariable Cox regression analysis by BRB‐Array Tools, we identified 85 and 21 ncRNAs that strongly correlated with the patients’ OS from the two sets, respectively. Among those transcripts, there were 11 overlapping ones and the lengths were more than 200 nucleotides. To make the model more practical, RSF were performed on the basis of the 11 lncRNAs, resulting in three lncRNAs remaining in the model. Therefore, a set of three lncRNAs was selected as the predictor for survival of GC (Table 1). Of these, *LINC01140* and *TGFB2‐OT1* showed a positive coefficient in the univariate analysis, indicating that their higher levels of expression were associated with a shorter survival. A negative coefficient indicated that patients with a higher level of expression of *RP11‐347C12.10* tended to have a longer survival compared with those with a lower expression. Table 1 also describes a list of these three genes with their obtained variable importance values, with *LINC01140* showing greater importance than the other predictors (Figure S1). All three lncRNAs have been verified in *LNCipedia* (a database for annotated human lncRNA sequences) and confirmed as ncRNAs in this website 18. Additionally, the noncoding nature of these lncRNAs was verified by coding potential analysis.

**Table 1:** LncRNAs significantly associated with the overall survival in GSE62254

Gene symbol	Permutation P value^a^^,^ b	Hazard ratio^a^	Coefficient^a^	Coefficient^c^	Variable importance	Relative importance
*LINC01140*	<1E‐07	3.877	1.35506	0.84321	0.0481	1.0000
*TGFB2‐OT1*	<1E‐07	4.102	1.41148	0.87302	0.0354	0.7363
*RP11‐347C12.10*	<1E‐07	0.004	−5.5215	−2.4496	0.0116	0.2414

a Derived from the univariate Cox regression analysis.

b Obtained from permutation test repeated 10,000 times.

c Derived from the multivariate Cox regression analysis.

### Three‐lncRNA signature and GC survival

**Figure 2:**
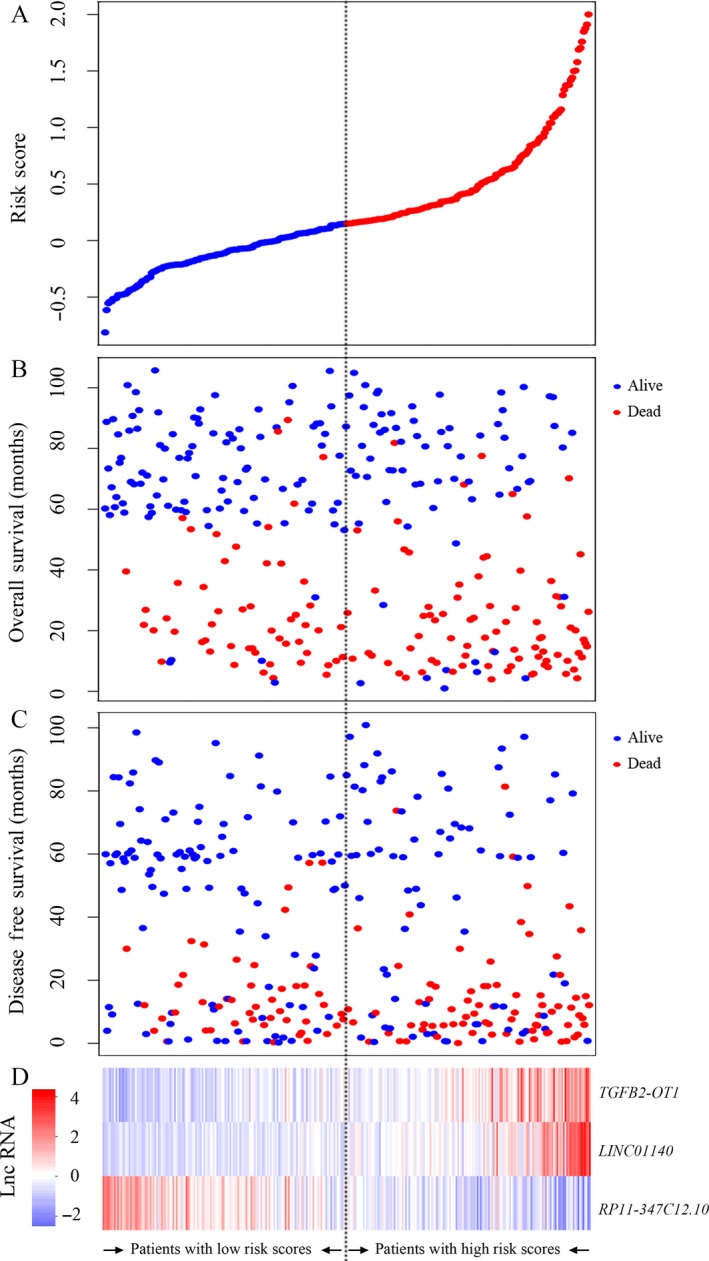
The distribution of the three‐lncRNA risk score, patients’ survival status, and lncRNA expression signature were analyzed in the GSE62254 set (*N* = 300). (A) LncRNA risk score distribution; (B) patients’ overall survival status and time; (C) patients’ disease‐free survival status and time; (D) heatmap of the lncRNA expression profiles. Rows represent lncRNAs and columns represent patients. The black dotted line represents the median lncRNA risk score cutoff dividing patients into low‐risk and high‐risk groups.

**Figure 3:**
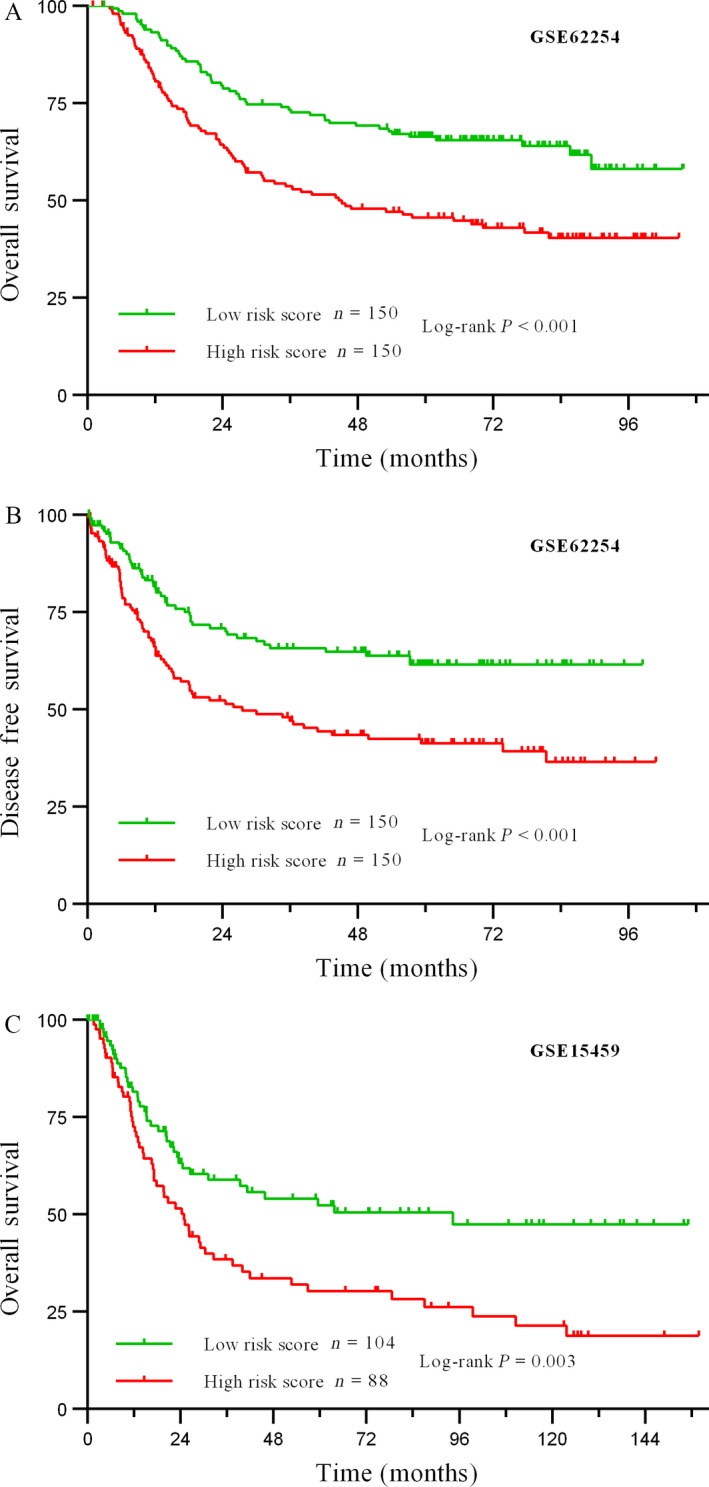
Kaplan–Meier estimates of the survival for patients using the three‐lncRNA signature. (A) Kaplan–Meier curves of overall survival for the GSE62254 set (*N* = 300); (B) Kaplan–Meier curves of disease‐free survival for the GSE62254 set (*N* = 300); (C) Kaplan–Meier curves of overall survival for the GSE15459 set (*N* = 192).

We created a risk‐score formula according to the expression of these three lncRNAs for OS outcome in the total GSE62254 set using multivariate Cox regression as follows: (0.84321 × expression level of *LINC01140*) + (0.87302*expression level of *TGFB2‐OT1*) + (−2.4496 × expression level of *RP11‐347C12.10*). The risk scores of the three‐lncRNA signature for each sample in the GSE62254 set were calculated and ranked according to the values. Figure 2 shows that patients with low‐risk scores tended to express high levels of protective lncRNAs (*RP11‐347C12.10*), whereas patients with high‐risk scores tended to express high levels of risky lncRNAs (*LINC01140* and *TGFB2‐OT1*). Using the median risk score (0.149) as the cutoff point, patients were divided into a high‐risk group (score >0.149, *N* = 150) and a low‐risk group (score ≤0.149, *N* = 150). As shown in Figure 3, we observed that GC patients with high‐risk scores had lower OS and DFS rates than those with low‐risk scores (both log‐rank test *P* < 0.001). To validate our findings, we classified patients in the GSE15459 set into a high‐risk (*N* = 88) and a low‐risk group (*N* = 104) by using the same cutoff value. Consistent with the findings described above, patients in the high‐risk group suffered significantly poorer OS than did those in the low‐risk group (log‐rank test *P* = 0.003).

### Stratified analysis of the three‐lncRNA signature and patients’ survival

To control for potential confounders and analyze phenotype‐specific survival, the patients were stratified by demographic characteristics and clinical features. As demonstrated in Table 2, we found that the decreased OS and DFS rates were noticeable for the patients with high‐risk scores among the subgroups of ≤64 years, >64 years, female, male, intestinal, diffuse, and noncardia in the GSE62254 set. Importantly, increased death was also pronounced for individuals with high‐risk scores among the subgroups of >64 years, male, diffuse, and stage III/IV in the GSE15459 set (adjusted HR = 1.84, 95% CI = 1.08–3.12 for >64 years; 1.93, 1.17–3.17 for male; 2.49, 1.18–5.24 for diffuse; 1.96, 1.24–3.12 for stage III/IV).

**Table 2:** Stratified analysis of the three‐lncRNA signature associated with gastric cancer patients’ survival

Variables	No. of patients	Univariate analysis			Multivariate analysis^a^		
		HR	95% CI	*P* value	HR	95% CI	*P* value
GSE62254 (OS)							
Three‐lncRNA risk score	300	1.93	1.36–2.72	<0.001	2.02	1.42–2.87	<0.001
Age							
≤64 years	161	1.98	1.17–3.34	0.010	2.02	1.19–3.41	0.009
>64 years	139	2.18	1.37–3.49	0.001	2.20	1.38–3.52	0.001
Sex							
Female	101	2.77	1.47–5.24	0.002	2.80	1.48–5.32	0.002
Male	199	1.62	1.07–2.45	0.024	1.76	1.15–2.69	0.009
Lauren							
Intestinal	146	1.94	1.13–3.32	0.017	2.07	1.20–3.56	0.009
Diffuse	134	1.75	1.03–2.95	0.037	1.81	1.06–3.06	0.029
Location							
Cardia	32	1.05	0.40–2.79	0.920	0.97	0.35–2.70	0.966
Noncardia	268	2.04	1.40–2.96	<0.001	2.12	1.45–3.09	<0.001
TNM							
I/II	126	1.60	0.79–3.22	0.189	1.71	0.84–3.46	0.139
III/IV	172	1.45	0.96–2.18	0.075	1.54	1.02–2.33	0.040
GSE62254 (DFS)							
Three‐lncRNA risk score	300	1.91	1.33–2.75	<0.001	2.00	1.38–2.89	<0.001
Age							
≤64 years	161	2.04	1.20–3.47	0.008	2.06	1.21–3.50	0.008
>64 years	139	2.05	1.24–3.40	0.006	2.05	1.24–3.40	0.005
Sex							
Female	101	2.59	1.37–4.93	0.004	2.67	1.39–5.11	0.003
Male	199	1.64	1.06–2.55	0.028	1.74	1.11–2.74	0.016
Lauren							
Intestinal	146	1.75	1.00–3.08	0.051	1.96	1.11–3.49	0.022
Diffuse	134	1.91	1.09–3.36	0.025	1.99	1.12–3.52	0.019
Location							
Cardia	32	1.08	0.40–2.91	0.873	0.94	0.33–2.62	0.901
Noncardia	268	2.07	1.40–3.06	<0.001	2.18	1.47–3.25	<0.001
TNM							
I/II	126	1.53	0.70–3.37	0.291	1.66	0.74–3.70	0.219
III/IV	172	1.40	0.93–2.12	0.111	1.48	0.97–2.27	0.069
GSE15459 (OS)							
Three‐lncRNA risk score	192	1.84	1.22–2.77	0.004	1.89	1.24–2.84	0.003
Age							
≤64 years	72	1.88	0.98–3.64	0.058	1.93	1.00–3.72	0.051
>64 years	120	1.82	1.07–3.08	0.027	1.84	1.08–3.12	0.024
Sex							
Female	67	1.61	0.77–3.35	0.204	1.68	0.80–3.53	0.175
Male	125	1.95	1.19–3.20	0.008	1.93	1.17–3.17	0.010
Lauren							
Intestinal	99	1.41	0.78–2.55	0.250	1.45	0.80–2.61	0.222
Diffuse	75	2.39	1.14–5.03	0.021	2.49	1.18–5.24	0.016
TNM							
I/II	60	2.6	0.80–8.47	0.113	2.35	0.71–7.81	0.163
III/IV	132	1.83	1.17–2.85	0.008	1.96	1.24–3.12	0.004

a Adjusted for age and sex.

HR, hazard ratio; CI, confidence interval; OS, overall survival; DFS, disease‐free survival.

### Correlation between the three‐lncRNA signature and clinicopathological features of gastric cancer patients

According to the median risk score, patients were equally classified into two groups (relative high‐risk group and low‐risk group). In the GSE62254 set, the differences in lncRNA risk scores were significantly associated with age (*P* = 0.049), histological types (*P* < 0.001), and TNM stage (*P* < 0.001). However, this significant correlation was only associated with histological types in the GSE15459 set (*P* < 0.001). When the risk score of the three‐lncRNA signature was evaluated in different strata of clinicopathological features, similar results were observed (Table 3).

**Table 3:** Correlation between three‐lncRNA signature and patients’ clinicopathological features

Variables	No. of patients	Three‐lncRNA expression			Three‐lncRNA score		
		Low *n* (%)	High *n* (%)	*P* value^a^	Mean (SD)	Median (IQR)	*P* value^b^
GSE62254							
Age				0.049			0.043
≤64 years	161	72 (44.7)	89 (55.3)		0.28 (0.53)	0.18 (−0.08–0.54)	
>64 years	139	78 (56.1)	61 (43.9)		0.16 (0.46)	0.10 (−0.13–0.36)	
Sex				0.714			0.160
Female	101	49 (48.5)	52 (51.5)		0.30 (0.57)	0.16 (−0.02–0.55)	
Male	199	101 (50.8)	98 (49.2)		0.19 (0.46)	0.15 (−0.13–0.41)	
Lauren				<0.001			<0.001
Intestinal	146	96 (65.8)	50 (34.2)		0.05 (0.37)	0.03 (−0.20–0.20)	
Diffuse	134	45 (33.6)	89 (66.4)		0.42 (0.56)	0.27 (0.06–0.69)	
Location				1.000			0.655
Cardia	32	16 (50.0)	16 (50.0)		0.23 (0.44)	0.15 (−0.04–0.53)	
Noncardia	268	134 (50.0)	134 (50.0)		0.23 (0.51)	0.15 (−0.12–0.42)	
TNM				<0.001			<0.001
I/II	126	81 (64.3)	45 (35.7)		0.07 (0.43)	0.01 (−0.21–0.21)	
III/IV	172	67 (39.0)	105 (61.0)		0.35 (0.52)	0.24 (0.01–0.59)	
GSE15459							
Age				0.231			0.060
≤64 years	72	35 (48.6)	37 (51.4)		0.42 (2.00)	0.26 (−0.87–1.88)	
>64 years	120	69 (57.5)	51 (42.5)		−0.14 (2.04)	−0.34 (−1.72–1.19)	
Sex							0.268
Female	67	33 (49.3)	34 (50.7)	0.317	0.32 (2.08)	0.21 (−1.29–1.70)	
Male	125	71 (56.8)	54 (43.2)		−0.07 (2.02)	−0.38 (−1.33–1.27)	
Lauren				<0.001			<0.001
Intestinal	99	68 (68.7)	31 (31.3)		−0.64 (1.76)	−0.51 (−1.89–0.39)	
Diffuse	75	26 (34.7)	49 (65.3)		1.06 (1.83)	0.86 (−0.47–2.61)	
TNM				0.639			0.431
I/II	60	34 (56.7)	26 (43.3)		−0.05 (2.38)	−0.27 (−1.62–1.50)	
III/IV	132	70 (53.0)	62 (47.0)		0.13 (1.88)	−0.02 (−1.00–1.30)	

a Statistical analyses were carried out using the chi‐square test.

b Statistical analyses were carried out using the Mann—Whitney *U* test.

IQR, interquartile range (from 25th percentile to the 75th percentile); SD, standard deviation; NA, not applicable.

### Stepwise Cox regression model for survival

A multivariate stepwise Cox regression analysis was performed to evaluate the correlation between variables, including selected demographic characteristics and clinical features, risk scores (as continuous variables) and GC survival. Finally, three variables (age, TMN stage, and three‐lncRNA risk score) of the GSE62254 set and two variables (TMN stage and three‐lncRNA risk score) of the GSE15459 set were included in the stepwise regression model (Table 4). Furthermore, multivariate stepwise models were applied in each data set based on selected variables. As a result, the variable of the three‐lncRNA risk score appeared in most stratified strata except the cardia, stage I/II subgroups in the GSE62254 set and the ≤64 years, female, intestinal, and diffuse subgroups in the GSE15459 set (Table S2).

**Table 4:** Results of stepwise Cox regression analysis on gastric cancer patients’ survival

Final variables	β	SE	HR	95% CI	*P* value
GSE62254 (OS)					
Age	0.03	0.01	1.03	1.01–1.05	0.001
TNM	0.78	0.11	2.19	1.77–2.72	<0.001
Three‐lncRNA risk score	0.89	0.16	2.42	1.79–3.28	<0.001
GSE62254 (DFS)					
Age	0.02	0.01	1.02	1.00–1.035	0.029
TNM	0.83	0.12	2.28	1.82–2.87	<0.001
Three‐lncRNA risk score	0.69	0.16	2.00	1.47–2.72	<0.001
GSE15459 (OS)					
TNM	1.04	0.14	2.82	2.16–3.68	<0.001
Three‐lncRNA risk score	0.15	0.05	1.16	1.04–1.28	0.005

β, regression coefficient; SE, standard error; HR, hazard ratio; CI, confidence interval; OS, overall survival; DFS, disease‐free survival.

### Identification of the three‐lncRNA signature associated with biological pathways and processes

**Figure 4:**
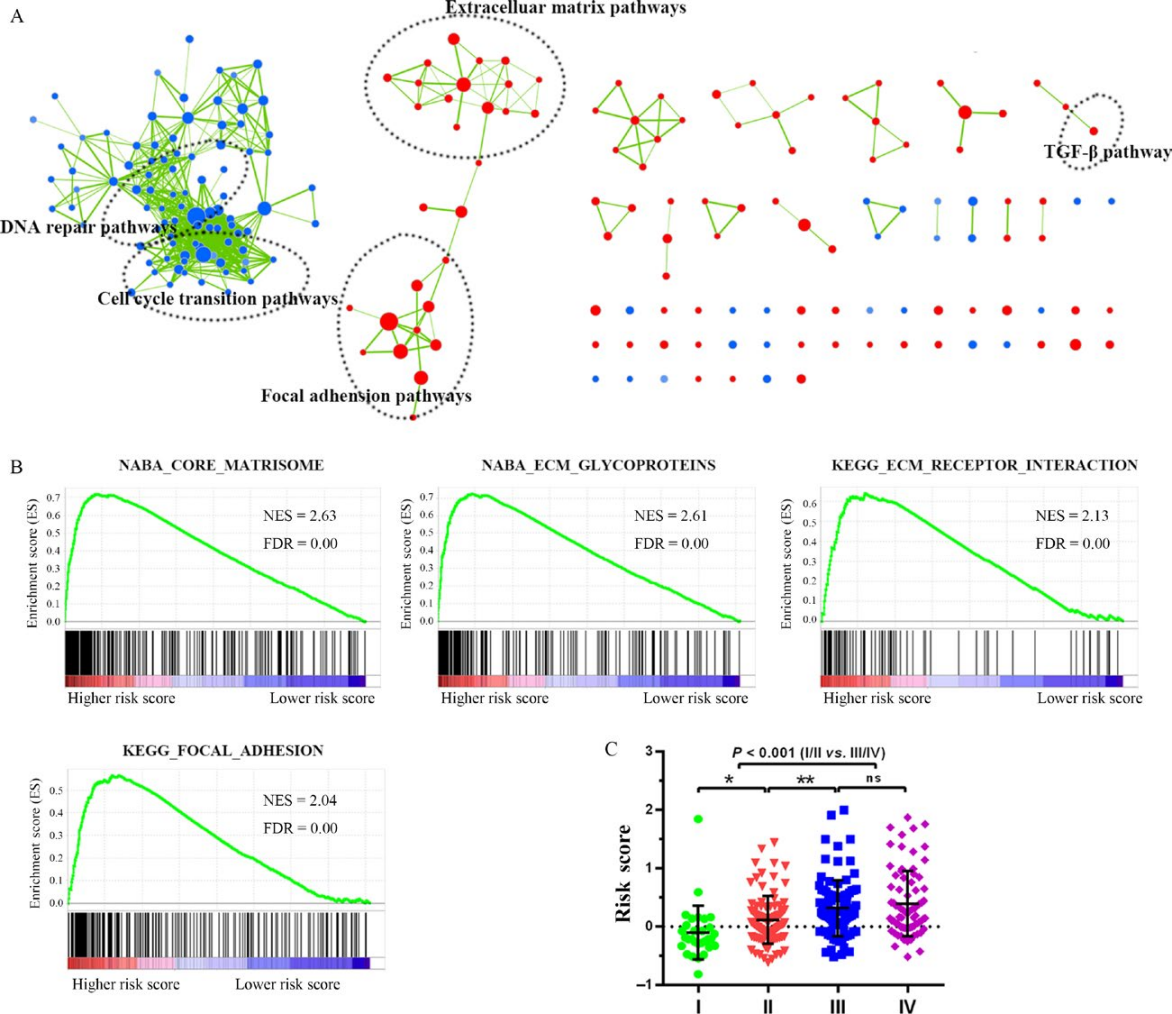
Performance evaluation of the three‐lncRNA signature in the GSE62254 set. (A) Gene set enrichment analysis delineates biological pathways associated with risk score using Cytoscape. Each node represents an enriched gene set, and they were grouped and annotated by the similarity according to related gene sets; (B) four typical cancer‐related pathways; (C) risk scores of patients with different TNM stages.

We carried out GSEA to identify associated biological processes and signaling pathways based on the risk score of the three‐lncRNA signature in the GSE62254 set (Table S3). The gene sets with a significantly different expression were visualized as interaction networks with the Cytoscape and Enrichment Map (Fig. 4A and B). Several cancer‐related networks, namely extracellular matrix pathways, integrin pathways, focal adhesion pathways and the TGF‐β pathway, were enriched in the high‐risk group, which implied that the signature might be involved in tumor metastasis. Thus, we compared the risk scores of patients with different TNM stages and found that patients at advanced stages tended to have higher risk scores than those at early stages (Fig. 4C, *P* < 0.001).

### Discriminatory and prognostic ability of the three‐lncRNA signature for survival

**Figure 5:**
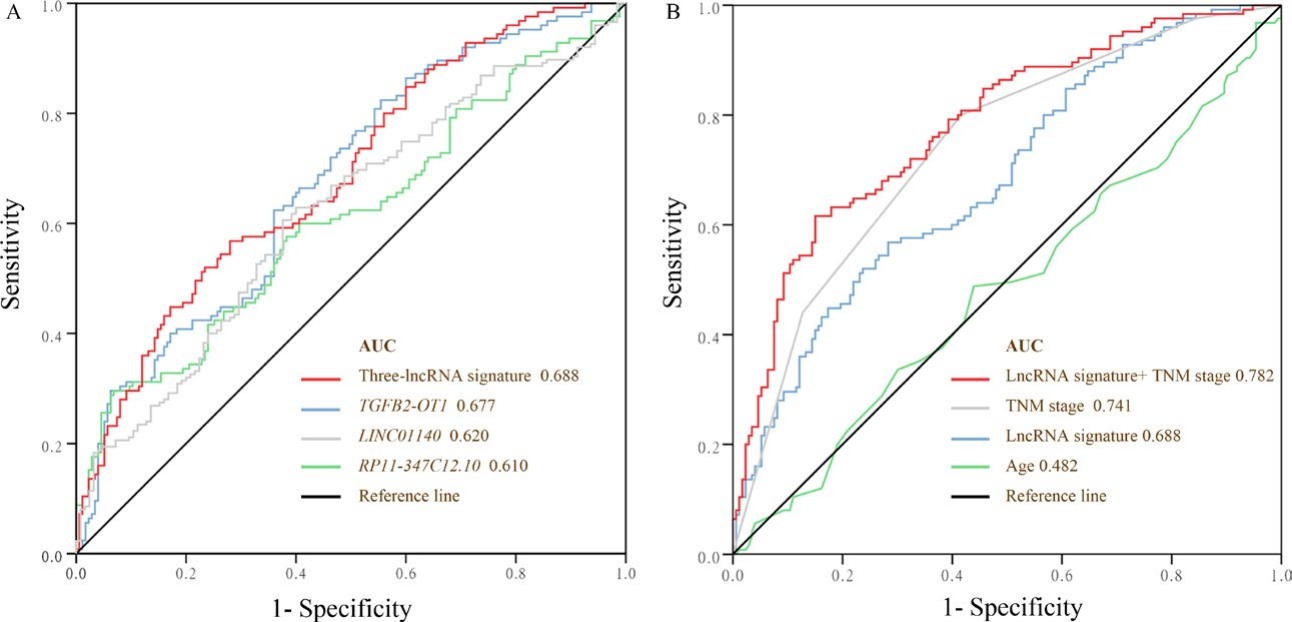
Receiver operating characteristic analysis of sensitivity and specificity by three lncRNAs, age, and TNM stage in predicting disease‐free survival in the GSE62254 set. (A) *LINC01140*,* TGFB2‐OT1*, and *RP11‐347C12.10*, and three‐lncRNA risk score; (B) age, TNM stage, three‐lncRNA risk score, and lncRNA risk score combined with TNM stage.

Because the GS62254 set contained the DFS information, we used ROC analysis to compare the sensitivity and specificity of GC recurrence between the risk score of the three‐lncRNA signature, TNM stage, and age of these patients. The area under the receiver operating characteristic (AUROC) was determined and compared among these three prognostic factors. Figure 5A shows that the AUROC of the three‐lncRNA risk score was 0.688, which was larger than that of each single lncRNA (0.677 for *TGFB2‐OT1*, 0.620 for *LINC01140*, and 0.610 for *RP11‐347C12.10*). In addition, as illustrated in Figure 5B, there was no significant difference between the AUROC of the three‐lncRNA risk score with the TNM stage (AUROC = 0.741, *P* = 0.187). However, we observed that the merged AUROC of three‐lncRNA risk score and the TNM stage (AUROC = 0.782) was larger than for each individually (for the three‐lncRNA risk score, *P* = 0.018; for the TNM stage, *P* = 0.301) and showed a good performance.

We also used a likelihood test to determine whether the signature really added consistent prognostic power to the TNM stage. Akaike information criterion (AIC) and Schwarz criterion (SBC) were employed to access the most appropriate model for OS in the GSE62254 set. The lowest value of AIC and SBC indicated the preferred model, which in this case was adopting both the three‐lncRNA signature and the TNM stage prognostic parameters (Table S4).

## Discussion

Over the past several years, a number of the genome's repertoire of nonprotein‐coding transcripts, including lncRNAs, has been viewed as inconsequential transcriptional “garbage.” Owing to the achievement of ENCODE and the implementation of The Cancer Genome Atlas (TCGA) program, lncRNAs have been highlighted for their important roles in cancer development and progression 19, 20. The involvement of lncRNAs in fundamental biological processes such as cell cycle regulation, apoptosis, and the DNA damage response and their implication in some human diseases are increasingly reported. More recently, most studies have shown that altered lncRNA expression levels are associated within the spectrum of disease development, but their prognostic values have rarely been investigated. To explore potential prognostic lncRNAs for GC, we achieved lncRNA profiling by mining the existing microarray gene expression data from the GEO database. In this present study, by analyzing the associations between lncRNA expression profiles and clinical features of GC patients in two large cohorts, a three‐lncRNA signature was identified that was significantly associated with patients’ OS and DFS.

By applying the three‐lncRNA signature to the patients of the GSE62254 and GSE15459 sets, obvious separations could be observed in the survival curves between patients with low‐ or high‐risk signatures. What was apparent from the results was that patients with low‐risk scores had a significantly prolonged survival time compared with patients with high‐risk scores. Regardless of whether the risk scores were evaluated as continuous variables or category variables, the correlation between this three‐lncRNA expression signature and survival was significant. In the stratified analysis, we further found that the three‐lncRNA risk score influenced the survival in different strata except for the cardia type, stage I/II, and stage III/IV subgroups for GSE62254, and female and stage I/II subgroups for GSE15459.

Further analysis uncovered that the lncRNA risk score was associated with age, Lauren type and TNM stage, especially the Lauren type. Compared with intestinal GC, diffuse GC has a greater tendency toward lymph node metastasis, advanced TNM stage, and a poor survival. In the diffuse GC patients, the risk scores of lncRNA were significantly higher than those in the intestinal GC patients. Despite the exact mechanism of the lncRNA signature with survival remaining unknown, the poor prognosis for patients with high‐risk scores could be partially due to its association with some key clinical characteristics (more aggressive pathological type and later TNM stage). Interestingly, even when stratified by some clinical variables (e.g., Lauren type), the prognostic value of the lncRNA signature still existed, which suggests that it might be a significant determinant of survival in GC and not an accidental feature of the transcription noise.

For the characteristics of the three lncRNAs, no functional studies involving them in GC were reported. To the best of our knowledge, our present study is the first to report the relationship between their expression levels and survival time. We then analyzed the genomic locations of these putative lncRNAs and found that they overlapped with some transcripts of oncogenes or tumor suppressor genes. *TGFB2‐OT1* is a newly discovered lncRNA derived from the 3′‐UTR of *TGFB2* and can regulate autophagy in vascular endothelial cells. Huang et al. reported that *TGFB2‐OT1* acts as a ceRNA, competes for binding with miR‐4459, miR‐3960, and miR‐4488, and regulates the expression of the miRNA targets to affect autophagy and inflammation 21. *LINC01140* and *RP11‐347C12.10* have been identified as long intergenic noncoding RNA, which may regulate the transcription of genomically neighboring protein‐coding genes in *cis* (such as *HS2ST1* and *CD2BP2*) and of distant protein‐coding genes in *trans* 22. Thus, it will be worthwhile to investigate the functional roles of these lncRNAs.

The identification of this three‐lncRNA signature that was associated with outcome in GC patients had some clinical implications. On the one hand, we found that the prognostic value of our three‐lncRNA signature was independent of age and TNM stage in the stepwise Cox regression. Presently, age and TNM stage, particularly the TNM stage, have been regarded as important predictors for survival of GC patients 23. Stage III and IV patients demonstrate a high local recurrence rate and poor survival outcome compared with those at stage I and II. However, clinically we can find that even patients with the same TNM stage might have different prognoses. This highlight the reasons for the unremitting exploration and hard work for identifying new biomarkers for a more precise survival prediction of high‐risk patients with GC and a consequently improved personalized cancer treatment. For this purpose, we developed a three‐lncRNA expression signature model that was closely associated with survival of GC patients of stage III/IV. However, this phenomenon was observed in the GSE15459 set and not in the GSE62254 set, so prospective multicenter large‐scale studies are essential to test the idea. On the other hand, the three‐lncRNA signature had a similar predictive value as the TNM stage for disease recurrence in the ROC analysis. The combination of the three‐lncRNA signature and the TNM stage could have a stronger power for DFS. The ability of our lncRNA signature implied that it could be useful for identifying subgroups of GC patients with identical TNM stages. Collectively, these data suggested that the three‐lncRNA signature might be a novel molecular target.

Moreover, GSEA was conducted to determine whether a predefined functional gene set showed coordinated expression based on the risk scores. The results of the GSEA revealed that the three‐lncRNA signature was more likely to involve extracellular matrix pathways, integrin pathways and focal adhesion pathways. The extracellular matrix can, via its receptors (especially the integrins), induce a variety of intracellular signals and regulate several cellular responses, including migration, differentiation, and proliferation, and has emerged as a major pathway contributing to cancer cell survival 24. Most notably, integrin‐mediated cell attachment has been shown to be required for tumor invasion and metastasis 25, 26. As is already well known, the major risk factors for GC prognosis are lymph node and distant metastasis, which usually occur in the advanced stages. Patients in the advanced stage were validated in our study to get higher risk scores than those in the early stage. Therefore, the enriched signaling pathways might support that our three‐lncRNA signature had survival prediction power and suggested possible avenues for future targeted therapies.

To date, gene expression profiling has commercially served as adjuncts for the treatment of cancers, including breast, prostate, and colon cancer. For example, the 21‐gene recurrence scores (Oncotype DX Breast Cancer Assay) are utilized as an important indicator to evaluate distant disease recurrence and the benefit of adjuvant chemotherapy in estrogen‐receptor‐positive breast cancer. However, no such effective prognostic tool is available for patients with GC to help physicians and patients determine the best course of therapy. As shown in this study, a small number of genes (three genes) could be sufficient to predict the prognosis of GC, which provide a valuable and feasible reference for clinicians.

Several limitations to this study needed to be noted. First, in our study only a portion of human ncRNAs was analyzed, and these ncRNAs were obtained by repurposing the microarray probes. Thus, the prognostic lncRNAs identified here might not represent all of the lncRNA candidates that potentially correlate with GC survival. Second, we did not investigate the mechanisms behind the prognostic values of these three lncRNAs in GC, and experimental studies on cancer cell lines and xenograft models would provide important information to further the understanding of their functional roles. Third, we only recapitulated our findings in two published datasets, and thus more datasets are required for further validation. It is worth mentioning that some important characteristics (age, TNM stage) between the two datasets are quite different, but the three‐lncRNA signature was still associated with patients’ survival in both two sets, indicating the prognostic value of this signature was robust.

In conclusion, our study reveals a three‐lncRNA signature that is linked to survival in GC patients. The prognostic value of this signature is independent of the TNM stage, one of the main predictive factors. Taken together, this innovative signature may serve as possible candidate biomarkers and therapeutic targets for GC. Future studies will focus on the validation of our findings in clinical trials and the functional effects of these identified lncRNAs.

## Conflict of Interest

The authors have declared that no competing interests exist.

## Supporting information


**Figure S1.** Random survival forests‐variable hunting analysis for identifying valuable lncRNAs. (A) Error rate for the data as a function of trees; (B) out‐of‐bag importance values for the three lncRNAs.

**Table S1.** Demographic and clinical features of including subjects involved in this study.
**Table S2.** Results of stepwise Cox regression analysis on gastric cancer patients’ survival among different groups.
**Table S3.** Gene set enrichment analysis describes biological pathways associated with risk score.
**Table S4.** Likelihood tests for different survival models.
