# Supplementary figures and images for: A three‐lncRNA expression signature associated with the prognosis of gastric cancer patients
Source: Cancer Med. 2017 Apr 26;6(6):1154–64. doi: 10.1002/cam4.1047 (PMC5463065; doi:10.1002/cam4.1047)

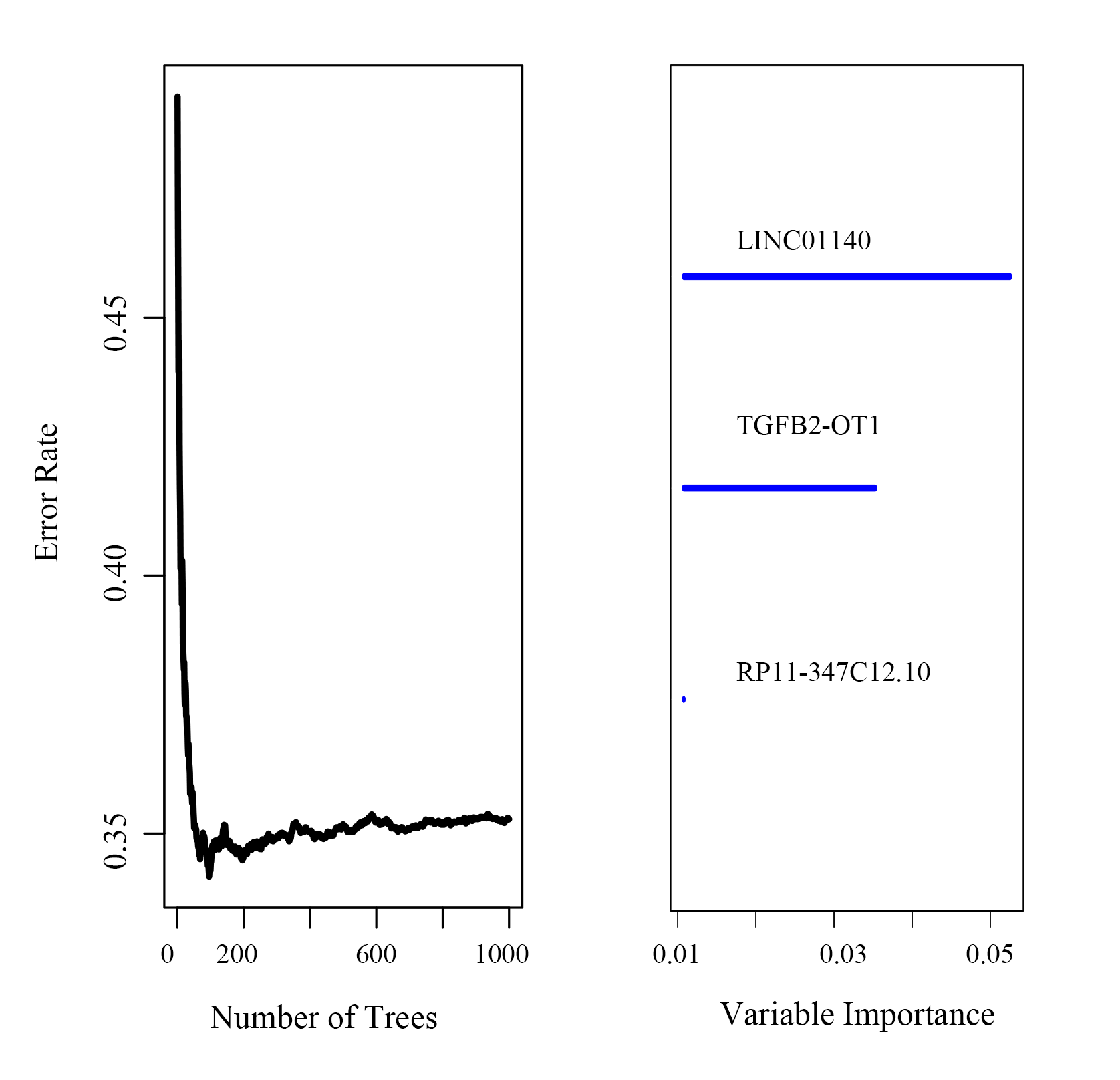

Supplement: Supplementary file 1 — Figure S1. Random survival forests‐variable hunting analysis for identifying valuable lncRNAs. (A) Error rate for the data as a function of trees; (B) out‐of‐bag importance values for the three lncRNAs. [file CAM4-6-1154-s001.tif]
