# Supplementary material for: A three‐lncRNA expression signature associated with the prognosis of gastric cancer patients
Source: Cancer Med. 2017 Apr 26;6(6):1154–64. doi: 10.1002/cam4.1047 (PMC5463065; doi:10.1002/cam4.1047)
Supplement: Supplementary file 2 — Table S1. Demographic and clinical features of including subjects involved in this study. Table S2. Results of stepwise Cox regression analysis on gastric cancer patients’ survival among different groups. Table S3. Gene set enrichment analysis describes biological pathways associated with risk score. Table S4. Likelihood tests for different survival models. [file CAM4-6-1154-s002.doc]

**Supplementary Table S1**. Demographic and clinical features of including subjects involved in this study

| Variables | GSE62254 (n = 300) | GSE15459 (n = 192) | P value.^a^ |
| --- | --- | --- | --- |
| Follow-up (month) |  |  |  |
| Mean (range) | 50.6 (1.0-105.7) | 38.4 (0.0-157.8) |  |
| Age (year) |  |  | 0.008 ^b^ |
| Mean±SD | 61.9 ± 11.4 | 64.4 ± 13.2 |  |
| Median (IQR) | 64.0 (55.0~70.0) | 66.6 (56.7~73.0) |  |
| Sex |  |  | 0.779 |
| Female | 101 (33.7%) | 67 (34.9%) |  |
| Male | 199 (66.3%) | 125 (65.1%) |  |
| Lauren |  |  | 0.191 |
| Intestinal | 146 (48.7%) | 99 (51.6%) |  |
| Diffuse | 134 (44.7%) | 75 (39.1%) |  |
| Mixed | 17 (5.7%) | 18 (9.4%) |  |
| Location |  |  | NA |
| Cardia | 32 (10.7%) | NA |  |
| Non-cardia | 268 (89.3%) | NA |  |
| TNM stage |  |  | < 0.001 |
| I | 30 (10.0%) | 31 (16.1%) |  |
| II | 96 (32.0%) | 29 (15.1%) |  |
| III | 95 (31.7%) | 72 (37.5%) |  |
| IV | 77 (25.7%) | 60 (31.3%) |  |
| Chemotherapy |  |  | NA |
| Yes | 80 (26.7%) | NA |  |
| No | 219 (73.0%) | NA |  |

^a^ Two-sided χ^2^test for the frequency distributions of selected variables between GSE62254 set and GSE15459 set.

^b^ Mann-Whitney U test.

IQR: interquartile range (from 25th percentile to the 75th percentile); SD: standard deviation; NA: not applicable.

**Supplementary Table S2**: Results of stepwise Cox regression analysis on gastric cancer patients’ survival among different groups

| Variables | GSE62254 (OS) | GSE62254 (DFS) | GSE15459 (OS) |
| --- | --- | --- | --- |
| Age |  |  |  |
| ≤ 64 years | TNM stage,  Three-lncRNA score | TNM stage,  Three-lncRNA score | TNM stage, |
| > 64 years | TNM stage,  Three-lncRNA score | TNM stage,  Three-lncRNA score | TNM stage,  Three-lncRNA score |
| Sex |  |  |  |
| Female | Age, TNM stage, Location, Three-lncRNA score | TNM stage,  Three-lncRNA score | TNM stage, |
| Male | Age, TNM stage,  Three-lncRNA score | TNM stage,  Three-lncRNA score | TNM stage,  Three-lncRNA score |
| Lauren |  |  |  |
| Intestinal | Age, Sex, TNM stage,  Three-lncRNA score | Age, Sex, TNM stage,  Three-lncRNA score | TNM stage, |
| Diffuse | Age, TNM stage,  Three-lncRNA score | Location, TNM stage,  Three-lncRNA score | TNM stage, |
| Location |  |  |  |
| Cardia | TNM stage | TNM stage | NA |
| Non-cardia | Age, TNM stage,  Three-lncRNA score | Age, TNM stage,  Three-lncRNA score | NA |
| TNM stage |  |  |  |
| I/II | No variables enter into the model | No variables enter into the model | Three-lncRNA score |
| III/IV | Age,  Three-lncRNA score | Three-lncRNA score | Three-lncRNA score |

NA: not applicable.

**Supplementary Table S4**: Likelihood tests for different survival models

| Model ^a^ | -2 Log(L) | AIC | SBC | Likelihood-ratio | P |
| --- | --- | --- | --- | --- | --- |
| Score | 1385.10 | 1391.10 | 1399.82 | 46.02 | < 0.001 |
| TNM | 1356.68 | 1362.68 | 1371.40 | 72.01 | < 0.001 |
| Score+TNM | 1327.86 | 1335.86 | 1347.48 | 100.83 | < 0.001 |

^a^ Including age and sex.

AIC: Akaike information criterion; SBC: Schwarz criterion.
